# Energy saving behavior in university organizations: The value of norm constructions in a “rational choice” action model

**DOI:** 10.3389/fpsyg.2023.1082061

**Published:** 2023-02-21

**Authors:** Sascha Heib, Jan Hildebrand, Timo Kortsch

**Affiliations:** ^1^Department of Environmental Psychology, Institute for Future Energy and Material Flow Systems (IZES gGmbH), Saarbrücken, Germany; ^2^Department of Health and Social Work, IU International University of Applied Sciences, Erfurt, Germany

**Keywords:** energy saving behavior, theory of planned behavior (TPB), social norms, university, structural equation modeling, identification

## Abstract

**Introduction:**

In times of rising energy prices and increasing importance of climate change, saving energy becomes even more important. Universities are large public institutions with significant energy-saving potential. This study investigated the energy-saving behavior of students and employees at a German university. In contrast to previous studies, which often focused on individual buildings, this study took a comprehensive approach and addressed all university members (employees and students). A extended model of the Theory of Planned Behavior (TPB) served as the theoretical basis. Considering the specific organizational context, the main question of the study was to explore the interlinkages between the intention to save energy, corresponding consumption behavior, and the effects of injunctive and descriptive social norms within the social system of the organization. Furthermore, the impact of “not energy related” factors like the identification with the organization was tested.

**Methods:**

As methodological approach a university-wide quantitative online survey was conducted. For the survey, a standardized questionnaire containing several scales on energy consumption behavior and TBP-constructs was used. All in all, data from a total of 1,714 members of the university participating in the study was analyzed.

**Results:**

Structural equation modeling showed that the extended TPB model yields a satisfactory amount of explained variance (approximately 40%) for intention and a moderate amount (approximately 20%) for behavior. The strongest predictors are personal norm and behavioral control. The organizational influence factor identification was also significant for intention, but only to a small extent.

**Discussion:**

The results extend the understanding of the TPB in the context of energy conservation in universities and emphasize that the sense of behavioral control and the personal norm must always be considered when intervening in this context to promote energy conservation and thus provide valuable hints for practical measures.

## 1. Introduction

A sustainable transformation of energy systems requires not only the conversion of energy supply systems to renewable energy generation, but also considerable measures on the demand side; i.e., energy consumption must be significantly reduced, and this must be done equally by government actors, commercial enterprises, organizations and each individual citizen (Wissenschaftlicher Beirat der Bundesregierung Globale Umweltveränderungen [WBGU], 2014). Since a large number of people can be reached immediately by means of interventions and since there is also great potential for energy savings and social multiplier effects, organizations represent important collective starting points–here, energy-sustainable organizational cultures can be developed, and models can be tested by way of example. How high the energy consumption is in an organization depends not only on structural aspects such as the heating system, but also on the actions of the individuals or members of the organization. The sum of individual energy-related *behavior* in the organizational context is a relevant factor that contributes to the success or failure of energy conservation efforts (Carrico and Riemer, 2011). For example, the savings potential through organizational measures and optimized user behavior in larger public (office) building complexes and universities is estimated to be about 10% (Matthies and Hansmeier, 2010). Based on the actual costs of a university of 5 million euros per year for electricity and heat, these savings mean that 500,000 € are not available for teaching expenses or other research-related measures. With regard to the current increase in energy prices and partly resulting measures such as online courses, it becomes clear how much pressure there is on the universities to reduce costs and how important it is to exploit the existing potential for reduction, both technically and in terms of behavior. Accordingly, in connection with usage behavior, it is important to create awareness of the issue, point out possible courses of action, and develop a conducive social norm.

Within the environmental psychological literature on energy saving, the majority of studies deal with the behavior of people in a private, domestic context (in summary, e.g., Abrahamse et al., 2005; Thøgersen and Grønhøj, 2010). In addition, there are studies that deal with the energy-related behavior of employees and members of company administrations, authorities, organizations, etc., institutions (e.g., Zhang et al., 2013; Lo et al., 2014; Dixon et al., 2015; Leygue et al., 2017). Recently, a meta-analysis was conducted on so called employee green behavior (EGB) (Katz et al., 2022) and an integrative conceptual model of EGB has been developed (Zacher et al., 2022). The influence and impact of many factors that determine energy-related behavior can change depending on the context or setting (e.g., home vs. workplace) (e.g., Scherbaum et al., 2008; Littleford et al., 2014). Of particular relevance in the workplace is, for example, the social environment, i.e., work colleagues and also supervisors and managers. It has been shown that they can exert a beneficial influence on environmentally relevant *behavior* (such as energy saving) at the workplace. This effect is based, among other things, on exemplary behavior and the motivation and support of employees in their efforts to save energy (e.g., Robertson and Barling, 2013). A number of studies also report positive correlations between organizational commitment and *identification* with the organization and environmental (protection) related *behavior* at the workplace (e.g., Lülfs and Hahn, 2013; Norton et al., 2015). These effects are attributed to an increased commitment to the organization’s goals and the tendency to adopt and internalize its values, norms, and beliefs (see Ashforth and Mael, 1989). However, in the organizational setting, negative group effects are also to be expected in the workplace, such as the diffusion or delegation of responsibility (e.g., Lo et al., 2014).

The sustainable use of energy at universities in particular has already received a certain amount of attention (e.g., Marcell et al., 2004; Schahn, 2007; Scherbaum et al., 2008; Marans and Edelstein, 2010; Carrico and Riemer, 2011; Matthies et al., 2011; Stumpf, 2014; Blok et al., 2015; Endrejat et al., 2017). However, in many of these studies only selected university buildings seem to serve as examples for office buildings in general. Compared to many other organizations (companies, public authorities, etc.), however, universities have some special features, e.g., a high degree of autonomy for decentralized institutions (faculties, chairs) and significant differences between their needs, possibilities, and willingness with respect to energy consumption and savings (Stumpf, 2014). Another characteristic of universities is the great heterogeneity of their members, e.g., in terms of role/function and duration of their membership in the organization. In this sense, universities are comparable to other complex public organizations such as hospitals (cf. Hildebrand et al., 2022). Only a few studies at universities include, for example, the diversity of building functions/uses (besides offices, including laboratories, lecture halls, libraries, refectories, etc.) and the various user groups (including the large group of students) in the investigations (e.g., Marans and Edelstein, 2010; Whittle and Jones, 2013; Chihib et al., 2021; Prafitasiwi et al., 2022; Rebelatto et al., 2022).

The present study deals with possible factors influencing the energy (saving) behavior of members of a medium-sized university as an example for the promotion of sustainable action in organizations from an environmental psychological perspective, based on an established action model. According to the assumptions of the Theory of Planned Behavior (TPB), behavior is directly determined by the behavioral intention, which in turn is influenced by attitudes toward the behavior in question, subjective norms related to the behavior, and perceived behavioral control (Ajzen, 1991). The latter is assumed to be able to exert a direct effect on behavior, bypassing the mediating intention (Ajzen, 1991; Ajzen and Fishbein, 2005). The TPB has repeatedly been criticized for failing to take sufficient account (as a rational choice model) of the importance of moral and altruistic behavioral drives (values, normative standards and obligations) for prosocial action, which includes environmental behavior (Bamberg and Schmidt, 2003; Klöckner and Blöbaum, 2010; Abrahamse and Steg, 2011; Gao et al., 2017). The only norm variable contained in the original TPB, the subjective norm (also referred to as the social norm in some studies), represents the social influence, i.e., the social pressure perceived by a person to exercise or not to exercise a certain behavior (Ajzen, 1991; Ajzen and Fishbein, 2005). These are norms that have their origin in the person’s social environment, not person-internal normative standards as represented by internalized (social) norms or personal/personal norms (Thøgersen, 2006; White et al., 2009; Bertoldo and Castro, 2016). The significance of the subjective/social norm as a co-determining factor for intention or behavior is not based on the fact that the person strives to meet his or her own moral standards or feels an inner obligation to behave in a normatively correct manner. The subjective/social norm therefore influences intention or behavior because the person wants to avoid punishment for socially undesirable (deviant) behavior and/or to experience rewards for socially desirable (norm-compliant) behavior (White et al., 2009; Bertoldo and Castro, 2016). The distinction between descriptive and injunctive social norms, which is established and widely used in social psychology (e.g., Cialdini et al., 1991; Göckeritz et al., 2010; Cialdini, 2012), seems to receive attention only occasionally and only recently in the context of TPB (e.g., Rivis and Sheeran, 2003; White et al., 2009; Gao et al., 2017). Descriptive social norms describe what is the “normal,” i.e., customary and majority behavior in a particular social environment. Behaving according to them is usually an efficient way for an individual to act effectively and adaptively in a given situation (Cialdini et al., 1991; Cialdini, 2012). In contrast, adherence to injunctive social norms is a way for an individual to experience social acceptance as it adapts to the moral rules of the group, since injunctive norms define what should be done in a community, i.e., what behaviors are approved or disapproved of Cialdini et al. (1991) and Cialdini (2012). In its original form, the construct subjective norm of TPB reflects mainly injunctive social norms, while descriptive social norms are not or hardly represented in it (Rivis and Sheeran, 2003; White et al., 2009).

A significant influence of social norms, both injunctive and descriptive, is particularly likely in cases where the social environment that is the source of these norms is composed of individuals or groups of people that are highly relevant to the acting individual. This relevance can arise, for example, from the fact that there are (more or less close) personal relationships with other people (e.g., family, friends, business) or that they form a social group to which one considers oneself a member (e.g., club, school class, company). In the context of occupational and organizational psychology, social identification/group identification is usually treated in the form of organizational identification (e.g., Ashforth and Mael, 1989; Van Dick, 2001). The assumption that the behavioral influence of social norms emanating from a particular social group depends on how strong an individual’s identification with that group is has been empirically tested several times in TPB models, including in the field of environmental behavior (e.g., Terry et al., 1999; Fielding et al., 2008; White et al., 2009; Nigbur et al., 2010). For example, in the context of the hotel industry, it was also shown that higher identification with the organization is positively related to the environmentally friendly behavior of employees (Shah et al., 2021).

In contrast to social norms, personal norms are not followed because they exemplify effective behavior (→ descriptive social norms) or because the violation of these norms is likely to result in negative sanctions of the social environment (→ injunctive social norms). Personal norms are followed in order to act in accordance with internal or internalized values and (moral) normative standards; the aversive consequences of non-compliance are also of internal origin, such as feelings of guilt, negative self-assessment, etc. (Thøgersen, 2006). Personal norms can thus be defined as self-expectations in relation to a specific action in a particular situation, which are experienced as a sense of moral obligation (Schwartz, 1977; quoted after Thøgersen, 2006). It is often assumed that the genesis of personal norms (as a rule) represents an internalization process of initially external, i.e., social (especially injunctive) norms, which are compared with existing, fundamental internal values and show themselves to be compatible with them (e.g., Thøgersen, 2006; Bertoldo and Castro, 2016). Some authors describe the interaction of the factors social norm and personal norm to explain concrete actions as follows: Social norms not only have a direct effect on behavioral intention, but also indirectly, mediated by personal norms (e.g., Bamberg et al., 2007; Bamberg and Möser, 2007; Klöckner, 2013). Thus, personal norms may only become a behavior-determining factor in a concrete situation once they have been activated by social norms (see Bertoldo and Castro, 2016).

These theoretical backgrounds and the reviewed literature leads to the following hypotheses:

Hypothesis 1: The energy saving intention of the university staff is predicted to a significant extent by the personal norm (H1a), injunctive (H1b) and descriptive social norm (H1c) and perceived behavioral control (H1d).

Hypothesis 2: The energy saving behavior is predicted by the energy saving intention (H2a) and perceived behavior control (H2b).

Hypothesis 3: The influence of the injunctive social norm on intention is at least partially mediated by the personal norm (H3a). This mediation effect is not expected with the descriptive social norm (H3b).

Hypothesis 4: The additional factor identification with the organization has a positive effect on energy saving intention.

Furthermore, building on hypotheses and findings from other authors, which point to a possible redundancy or substitutability of the constructs attitude and personal norm (e.g., Kaiser, 2006; Chan and Bishop, 2013) we want to examine if both constructs could be separated or can be used interchangeable and should be integrated to one construct.

RQ1: Are the constructs attitude and personal norm separable?

## 2. Materials and methods

### 2.1. Data collection and study population

The research was part of the larger project “Energiemustercampus UdS–Liegenschaftsweite Energiever–brauchsoptimierung (EULE)” (Energy model campus UdS–property-wide energy consumption optimization) funded by the German Federal Ministry for Economic Affairs and Climate Action (FKZ: 03ET1060A). The data collection *via* online questionnaire took place in November 2013. The population addressed by this study consisted of all members of Saarland University (UdS), i.e., all staff members (approx. 2,800) and all students (approx. 18,300) which were contacted *via* a central email distribution list, informed about the study and given link access to the questionnaire. Before starting the survey, the questionnaire was approved by both the scientific and as well as by the non-scientific staff council of the university. The main distribution channel was the general email distribution list of the university. Thus, both employees and students could be reached with one email. After the initial mail, there was 4 weeks later a reminder *via* mail. In addition, a link was placed on the university’s Facebook page to better reach students. Furthermore, the survey was advertised on information screens, for example in the cafeteria. The overall response rate was 9% (students 6%, employees 26%).

### 2.2. Questionnaire and instruments

The questionnaire comprises a total of 74 content-related questions (71 items in the format of rating scales with different numbers of levels plus three open questions), 12 questions on demographic data, and a field for comments. The questionnaire covers a variety of constructs from different models of environmental awareness and behavior, which have been operationalized partly through self-designed scales, partly through (adapted) scales that have already been empirically tested and established. For the present study, however, only a selection of the constructs was considered.

These include as independent variables: personal norm (as a possible substitute for attitudes), injunctive social norm, descriptive social norm, behavioral control, identification. Dependent variables are energy saving intention and energy saving behavior. Concerning energy saving behavior, the items which were included in the questionnaire are related to power consumption (use of electrically operated devices) and heating behavior (including ventilation and the like, as well as hot water) and only every day or at least with high regularity executed user behavior was asked, not purchase or investment behavior. The item list for these variables is displayed in the Supplementary material. Important characteristics and properties of the scales formed are shown in Table 1. The reliability statistics are all in the acceptable to very good range with α = 0.77 for energy saving behavior to α = 0.91 for Injunctive Social Norm. Only the value for the attitude scale is below convention at 0.44 (intercorrelation, since the scale consists of only two items).

**TABLE 1:** Descriptives of the included scales.

Scale	Items	Value range	Cronbach’s α^1^	M	SD
Attitude	2	1–5	0.44	4.20	0.71
Personal norm	3	1–5	0.84	4.43	0.56
Injunctive social norm	2	1–5	0.91	2.76	1.03
Descriptive social norm	2	1–5	0.84	2.77	0.82
Behavioral control	7	1–5	0.85	3.11	0.77
Identification	6	1–5	0.88	2.62	0.90
Energy saving intention	2	1–5	0.90	3.99	0.82
Energy saving behavior	9	1–6	0.77	4.45	0.97

M = mean value, SD = standard deviation.

^1^If the scale consists only of two items, the bivariate correlation is reported instead of Cronbach’s alpha.

### 2.3. Sample

A total of 1,714 participants formed the final sample. Concerning gender, 74 participants did not provide any information, 54% of the others were women and 42% men. The mean age is 30.3 years (*SD* = 11.7). The average length of membership of UdS is 6.4 years (*SD* = 8.4). A total of 56% of the participants are students, 21% employees with scientific work, and 16% employees in the non-scientific area, for 7% information is not available.

### 2.4. Data analysis procedure

For statistical analyses we used R (R Core Team, 2018) with the lavaan package (version 0.6-6, Rosseel, 2012) and applied a two-step procedure. First, with confirmatory factor analyses we investigated the separability of the included scales. In this regard, we compared two structural models (one model assuming personal norm and attitude to be one factor and one model assuming personal norm and attitude to be two separable factors, see section “5. Conflict of interest” for the discussion of the separability of personal norm and attitude) (hypothesis 2). Second, a structural equation model was computed to investigate the direct (hypotheses 1 and 4) and indirect effects (hypothesis 3) which were assumed in the hypotheses. For stronger statistical inferences, we used the option “’bootstrap” (*N* = 1000) to bootstrap the estimates of the standard errors.

## 3. Results

### 3.1. Separability of the constructs and common method variance

**FIGURE 1:**
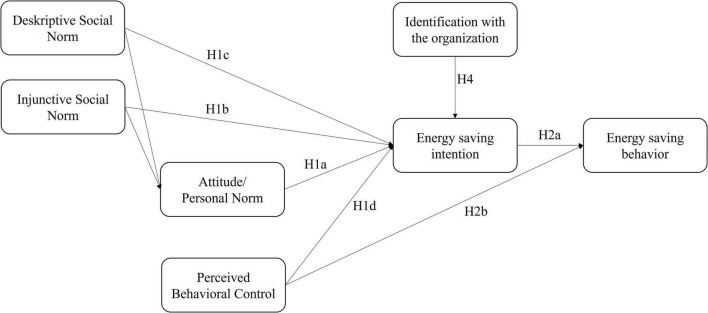
Research model and hypotheses. The mediation hypotheses H3a and H3b are not displayed in this figure.

To show the validity and separability of all included constructs (cf. Figure 1), we performed two confirmatory factor analyses. Furthermore, with this analysis we will investigate the research question RQ1 that asks whether attitude and personal norm are separable constructs. A model with seven factors (model 1; i.e., descriptive social norm, injunctive social norm, attitude = personal norm, behavioral control, identification, intention, behavior) assuming personal norm and attitude to be one factor and one model with eight factors assuming personal norm and attitude to be two separable factors (model 2; i.e., descriptive social norm, injunctive social norm, attitude, personal norm, behavioral control, identification, intention, behavior) were compared statistically.

Model 1 (X^2^ = 1980.82, *df* = 474, *p* < 0.001, *CFI* = 0.92, *RMSEA* = 0.05, *SRMR* = 0.04) and model 2 (X^2^ = 1861.69, *df* = 467, *p* < 0.001, *CFI* = 0.93, *RMSEA* = 0.05, *SRMR* = 0.04) had both a good fit (cf. Hu and Bentler, 1999). The fit of model 2 was superior to model 1 (ΔX^2^ = 19.13, Δ*df* = 7, *p* < 0.001). However, there was a nearly perfect latent correlation between attitude and personal norm (ρ = 0.93, see Table 2) which can also lead to multicollinearity problems. Thus, although it is not entirely clear concerning research question RQ1, the evidence was more in favor of combining the factors because model 1 (which assumes personal norm and attitude to be one factor) had a good model fit and personal norm and attitude had a nearly perfect latent correlation. Therefore, we decided to combine attitude and personal norm items to one factor attitude/personal norm for the further analyses.

**TABLE 2:** Manifest (below the diagonal) and latent intercorrelations (above the diagonal) of the included scales.

	1	2	3	4	5	6	7	8
1. Attitude		0.93	0.33	0.01^ns^	0.52	0.13	0.72	0.40
2. Personal norm			0.28	0.05^ns^	0.39	0.11	0.53	0.28
3. Injunctive social norm		0.27		0.28	0.30	0.21	0.33	0.21
4. Descriptive social norm		0.03^ns^	0.27		0.20	0.28	0.15	0.09
5. Behavioral control		0.38	0.26	0.17		0.22	0.54	0.40
6. Identification		0.10	0.18	0.24	0.22		0.19	0.12
7. Energy saving intention		0.53	0.28	0.13	0.46	0.19		
8. Energy saving behavior		0.30	0.19	0.10	0.34	0.14	36	

If not marked with “n.s.” (not significant) all correlations *p* < 0.001.

To assess the problem of common method variance, we also calculated Harman’s one-factor test (Harman, 1976), which assumes that results are threatened by common method variance when a single factor accounts for more than 50% of the total variance. The calculations showed that a single factor accounted for 19.1% of the total variance, which argues against the presence of common method variance in the data.

### 3.2. Results of the structural equation model

A structural equation model was computed using the *full information maximum likelihood* procedure which handles missing values as good as imputation procedures (Collins et al., 2001). However, there were *N* = 123 missing patterns which could not be handled because of too little information.

**FIGURE 2:**
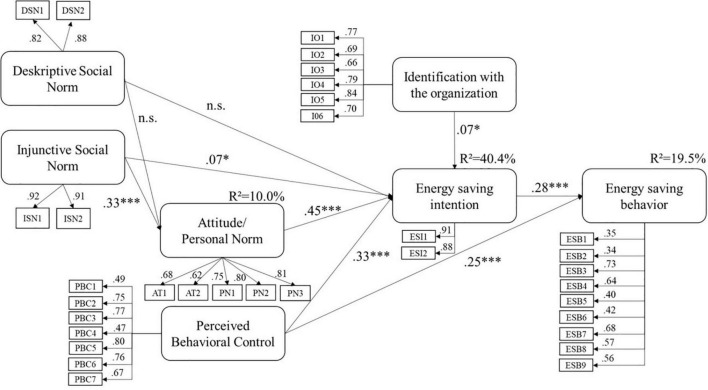
Results of the structural equation modeling. Significance levels: *p < 0.05, ***p < 0.001, n.s. not significant.

The bootstrapped model (*N* = 1000) fitted the data well (X^2^ = 2425.98, *df* = 480, *p* < 0.001, *CFI* = 0.92, *RMSEA* = 0.05, *SRMR* = 0.07). Consistent with our hypotheses, the intention to safe energy is significantly predicted by the factors attitude/personal norm (β = 0.45, *p* < 0.001; hypothesis H1a), injunctive social norm (β = 0.07, *p* = 0.023; hypothesis H1b), perceived behavioral control (β = 0.33, *p* < 0.001; hypothesis H1d) and identification (β = 0.07, *p* = 0.014; hypothesis H4). The following applies to all of these factors: the higher their value, the greater the intention to save energy. On the other hand, and contrary to the hypothesis, the factor descriptive social norm does not predict energy saving intention (β = 0.04, *p* = 0.194; hypothesis H1c). In line with the hypotheses, and with the TPB, intention (β = 0.28, *p* < 0.001; hypothesis H2a) and behavioral control (β = 0.25, *p* < 0.001; hypothesis H2b) are significant predictors of energy saving behavior (see all results in Figure 2).

Concerning the hypothesized indirect effects of social norms on intention, the analyses revealed that the path from injunctive social norm *via* attitude/personal norm on intention was significant (β = 0.15, *p* < 0.001). This supports hypothesis H3a. As expected in hypothesis H3b, the corresponding path from the descriptive social norm was not significant (β = −0.02, *p* = 0.209).

The model accounts for 40.4% of the variance of energy saving intention and for 19.5% of the variance of energy saving behavior.

## 4. Discussion

The model of an extended TPB designed in the context of this study seems to be basically suitable to describe and explain energy-related intentions and corresponding behavior in the considered organizational context (i.e., an university). The intention is subject to a strong direct influence of the factors personal norm and behavioral control, a less strong influence of the injunctive social norm and no significant influence of the descriptive social norm. In addition, the (organizational) identification also contributes to the prediction of the energy saving intention. Behavior is essentially determined by behavior control and intention. To a lesser extent, the personal norm also exerts a direct influence on behavior, without mediation *via* the intention. Although this finding contradicts the assumptions of the TPB, it confirms the results of several other studies (e.g., Bamberg et al., 2007; White et al., 2009).

In the following, the results are first discussed in terms of their significance for research and theory building, before implications for energy conservation practice in institutions of higher education are derived, and finally some limitations and future research directions are discussed.

### 4.1. Theoretical implications

The attitude/personal norm factor was the strongest predictor for intention prediction, even before behavioral control. Previous studies that introduced personal norms into the TPB model usually did so as an additional factor and only rarely as a substitute for attitudes. We tested two alternative models, one with a personal norm as a substitute for attitudes and one that includes both factors. The results of the structural equation modeling showed marginal advantages of the model with both factors based on the fit characteristics, but the nearly perfect latent correlation speaks more for the combination of the factors. A meta-analysis showed the following: When personal norms are included in the prediction in addition to the other TPB constructs, personal norms contribute about as much to intention prediction as attitudes (Bamberg and Möser, 2007), and in some cases significantly more (e.g., White et al., 2009). According to Harland et al. (1999), personal norms as an additional factor increase the explanatory power of TPB in different environmental behavioral areas, which is associated with a decrease in the explanatory contribution of the factor attitude. This already indicates that there may be overlaps between the personal norm and attitude constructs (see also Thøgersen and Ölander, 2006). The extent of this overlap is the subject of controversial debate. Views that the two constructs have little or no discriminatory validity and are (more or less) interchangeable within the TPB (Kaiser, 2006; Chan and Bishop, 2013) have been criticized: Botetzagias et al. (2015), for example, came to different conclusions in their study. The model that best matched their empirical data showed personal norms as a strong predictor of attitudes, but still the greater part of their influence on intentions was direct, not mediated through attitudes. Klöckner (2013), on the other hand, came to findings that support the assumption that the influence of personal norms on intentions is mainly mediated by attitudes to the behavior in question. According to him the assessment of whether a behavior conforms to personal values is included in the overall assessment of whether this behavior is beneficial. In some cases, the attitude toward environmentally related behavior even seems to be strongly dominated by personal moral-normative considerations (see Thøgersen and Ölander, 2006). A study, which also addressed the issue of energy saving at the workplace, and in which the two constructs personal norm and attitude were considered together in a TPB model, yielded inconsistent results (Lo et al., 2014): In only two of four behaviors studied was the personal norm a significant predictor of the corresponding intention, and its predictive contribution in each case lagged (significantly) behind that of the attitudes. All this taken together leads to the assumption that the relevance of personal moral-normative evaluations for the intention to behave in an environmentally friendly manner varies depending on the specific behavior in a concrete situation. The internal moral influence seems to be different for energy saving (e.g., Lo et al., 2014) than for mobility behavior (e.g., Bamberg et al., 2007) or recycling (e.g., Botetzagias et al., 2015). And even for different behaviors within a domain, the importance of personal norms varies (see for energy saving Botetzagias et al., 2014).

In addition to the personal norm, two forms of social norms were examined in this study with regard to their influence on intention and behavior with respect to energy saving. For the prediction of intention, the injunctive social norm proved to be a significant predictor with an independent, albeit small, predictive contribution. In contrast, the descriptive social norm did not make an independent contribution to the prediction of intention. This finding pattern is opposite to that of the studies by White et al. (2009) and Nigbur et al. (2010), which, however, investigated recycling behavior. A meta-analysis by Rivis and Sheeran (2003) also found descriptive social norms to be the stronger predictor (compared to subjective norms, which are mainly of an injecting nature). However, as other authors have done, Thøgersen (2006) points out that the influence of different norm constructs can vary in strength depending on the environment-related behavior involved. For example, in studies investigating energy savings in the home environment, a low to absent influence of social norms does not seem unexpected, since in this context behavior is hardly exposed to visibility to other people, and thus no sanctions are threatened if the norm is violated (Botetzagias et al., 2014). In part, this may also apply to the energy-saving behavior at the university examined here, depending on the specific behavior and the spatial setting (e.g., individual office). In general, however, behavior at the university, as a public space, is more exposed to observation by others, which suggests a greater influence of social norms on energy saving behavior (on the effect of the number of colleagues at the workplace, see Lo et al., 2014). Social norms–especially descriptive ones–should be more present and salient here through observation of the behavior of fellow humans than in the private sphere of the home (Botetzagias et al., 2014; Blok et al., 2015).

As became apparent with the help of indirect effects analyses, in the present study the influence of the injunctive social norm on the intention was partly indirect, mediated *via* the personal norm. The size of the indirect effect corresponds approximately to that of the direct effect. In accordance with the hypotheses, such an indirect effect could not be demonstrated for the descriptive social norm. The role of personal norms as a mediator between social norms and intentions has already been addressed and empirically investigated several times (e.g., Bamberg et al., 2007; Klöckner and Blöbaum, 2010; Nigbur et al., 2010; Bertoldo and Castro, 2016).

If one follows the view that personal norms and injunctive social norms share the injunctive character (see Thøgersen, 2006), which means that both are associated with concepts of the type “good vs. bad” (in the moral sense), whereas descriptive social norms tend to stand for the evaluation dimension “right vs. wrong” (in the functional sense), it seems plausible that the mediation effect is primarily evident in the injunctive social norm. Nigbur et al. (2010) also report that mediation by personal norms only concerns the influence of injecting social norms on intentions, but not that of descriptive social norms. However, in that study (in contrast to the present study) the descriptive social norm was a significant direct predictor of intention, while the injunctive social norm was not. This leads the authors to conclude that both social norms are significant influencing factors, but differ in their mode of action. Bertoldo and Castro (2016) found that descriptive social norms are generally better suited to predict personal norms than injunctive social norms. However, when the strength of identification with the reference group that predicts social norms is taken into account, the picture is less clear. The effect of descriptive social norms on personal norms is largely independent of the measure of identification, whereas the effect of injunctive social norms on personal norms is in turn moderated by identification. In concrete terms, this means that personal norms are more strongly influenced by descriptive social norms in persons who do not identify strongly with the reference group; when identification with the group is strong, injunctive social norms exert a greater influence on personal norms (Bertoldo and Castro, 2016).

This leads the discussion directly to organizational identification. This proved to be a significant variable in predicting the energy saving intention. As an independent predictor, a high degree of identification with the UdS promotes the intention. This finding is consistent with a number of studies that report positive correlations between loyalty to a company or identification with an organization and environmental (protection) related behavior at the workplace (e.g., Lülfs and Hahn, 2013; Norton et al., 2015). No statements on the mechanism of action can be made here. In the literature these effects are explained in different ways. For example, the impact of identification on behavior is attributed to an increased commitment to the organization’s goals and a tendency to adopt its values, norms and beliefs (see Ashforth and Mael, 1989). Or, the effect results from the fact that a stronger identification increases the motivation to contribute to a good organizational image, which in turn positively influences (energy-saving) behavior (see Leygue et al., 2017).

The effect of identification, however, took place to a greater extent in the form of an interaction effect; identification acts as a moderator variable that changes the strength of the influence of social norms on the intention. This moderating effect of identification occurred in both the injunctive social norm and the descriptive social norm. This is an interesting finding, because previous studies have often found such moderation for heterogeneous norm constructs that combine injunctive and descriptive content (e.g., group norm in Terry et al., 1999; and perceived group norm in White et al., 2009). Thus, it was initially left open whether the effect can also be expected for purely descriptive social norms and/or purely injunctive social norms. Nigbur et al. (2010) tested in two studies the influence of injunctive and descriptive social norms on (recycling) intentions and behavior separately by means of a (neighborhood) identification measure. As the authors report, no moderation effect was found for the injunctive social norm, while in the case of the descriptive social norm there was at least some evidence for one; the descriptive social norm could therefore have a stronger influence on recycling behavior in persons who show a low level of identification with their neighborhood. In the same direction, the moderating influence of identification was shown in the present study, both for the descriptive and the injunctive social norm. A stronger identification with the university has the consequence that social norms have less influence on energy saving intentions. The direction in which identification here changes the relationship between social norms and intention does not follow the pattern known from other studies (Terry et al., 1999; White et al., 2009). In the above-mentioned studies, the (positive) influence of social norms on environmental behavior (or intentions) was greater in persons who identify more strongly with the group than in persons who have low group identification. We can only speculate here about an explanation for the finding that deviates from this pattern. It would be conceivable that identification with the university and social norms do not behave additively in their effect on energy saving intentions, but rather compensatory. In the case of persons who identify strongly with the university, it may be that the mere fact that this identification is of great importance for their social identity (as members of the UdS) leads them to behave in a manner that is in keeping with the organization in terms of energy use, regardless of how clearly they perceive the prevailing social expectations and practices with regard to this topic. More important than the general adherence to social norms seems to be acting out of loyalty and a sense of duty to the university. For individuals who do not feel a strong identification with the university, the extent to which they perceive injunctive and descriptive social norms plays a greater role in the intention to behave in an energy-saving manner. If the expectations and the practice exemplified by the environment visibly prescribe it, action is taken accordingly; but then presumably without thinking about the possible costs or benefits of the action for the organization.

The strong beneficial influence of behavioral control, which is perceived as a major factor, is consistent with other findings from the environmental psychological field of application of the TPB. Behavioral control often proves to be one of the best predictors of environmentally relevant behavior or corresponding intentions, including energy saving (e.g., Botetzagias et al., 2014; Ahmad et al., 2022, recycling) (e.g., White et al., 2009; Chan and Bishop, 2013; Botetzagias et al., 2015) and mobility behavior (e.g., Harland et al., 1999). However, there are also studies that report that this factor is less important, especially for environmental behavior in the workplace and especially in the domain of energy use (e.g., Greaves et al., 2013; Lo et al., 2014; Blok et al., 2015). Lo et al. (2014) found a varying degree of (positive) influence of perceived behavioral control for different behaviors at office workplaces associated with energy use. The authors cite an interaction effect with the number of office colleagues (and related shared responsibility or responsibility diffusion), which varies in strength depending on the behavior, as a possible explanation.

Overall, it can be said that according to the extended model of the TPB, personal moral-normative motivations on the one hand and assessments of practical feasibility on the other hand are decisive determinants for the individual energy-saving behavior of UdS members. In contrast, the orientation of one’s own actions to expectations and (informal) rules of the social environment is less important. The same applies to the degree of perceived identification with the UdS as a potential part of the social identity of its employees and students. In this context, the results of this study suggest that it might be worthwhile to take a closer look at questions concerning the respective reference group in terms of social identity: Which social group functions as the source of social norms, what exactly do UdS members identify with? With the university as a whole, with a faculty, with a small group of fellow students or work colleagues?

### 4.2. Practical implications

The results of the study provide besides the discussed theoretical implications also some ideas for designing practical intervention measures to promote energy-saving behavior at the university. The generally high level of personal norms with regard to energy saving among university members is a favorable prerequisite and gives rise to the hope that corresponding measures will fall on fertile ground. Interventions could aim to recall these latent personal norms in an organizational context and activate them in a situation-specific way (see Lülfs and Hahn, 2014). Potential measures on the personal norm level could be increasing knowledge concerning interrelations between different behavior styles and possibilities of action *via* awareness raising workshops and the provision of information material. Such workshops on green training topics strengthen the commitment to green behavior of employees the more they feel the obligation to take care of the organization (in this sense, they can be understood as a personal norm; Paillé and Valéau, 2021). Furthermore, direct instructions or prompts and reminders (stickers, posters, signs) for initiating behavior in the specific working place situation are effective measures. Additionally, individual self-commitment and target agreements combined with feedback systems for visualizing the effects of the consumption behavior in order to reinforce and maintain energy saving behavior are potential measures which are strongly related to the personal norm (Carrico and Riemer, 2011).

Since personal norms can be activated by (injunctive) social norms (e.g., Bertoldo and Castro, 2016), they should also be made salient in other ways–e.g., by influential persons within a faculty/work group or department (see Robertson and Barling, 2013). A combined use of injective and descriptive standards is considered particularly promising (Cialdini, 2012). It is therefore recommended that students and staff throughout the university be made aware that energy-efficient or generally sustainable action is a matter of course at the university, at all organizational levels. In this respect, addressing the social system in a broad approach could be an energy saving day at the university and a series of lectures with presentations on energy, in order to raise awareness. Here, the transfer from the general level to the individual level described above is relevant. For this transfer, multipliers could be trained to exemplify such behavior in their social environment at the university within a peer to peer approach. In addition, approaches using social competition in terms of energy savings between groups or departments is one measure which relates to social norms and social identification. Of course, as also indicated in this study, the combination of the different levels might be the most powerful strategy.

However, besides behavioral measures, more cost-intensive structural and technical measures would also be required to make it clear that the university is committed to creating structural conditions for the development of a sustainable energy culture within the organization (see Whittle and Jones, 2013). This clear communication of the university’s own sustainability goals and strategy might even have a positive effect on strengthening the identification with the organization as this illustrates the responsibility and active shaping of the future of the organization. For this purpose, it can be helpful to develop a corporate social responsibility strategy, as this additionally strengthens identification with the organization (Shah et al., 2021).

### 4.3. Limitations and future directions

Finally, a few limitations of this study must be pointed out. Since the results are based exclusively on data collected by means of a questionnaire, the possibility of a method effect must be pointed out (common method variance; see Podsakoff et al., 2012; Greaves et al., 2013). Even though the result of Harman’s one-factor test indicated against the threat of common method variance to the results, future studies should choose a study design that avoid this problem (e.g., a longitudinal design). Furthermore, the question about the separability of attitude and personal norm could not be answered definitively in this study, as the statistical results were inconclusive in this regard. In this respect, the results fit the previous presented broad scientific discussion (cf. Kaiser and Scheuthle, 2003; Kaiser, 2006; Chan and Bishop, 2013). Consequently, this question and the used scales should be re-examined in future studies in combination with other well-established instruments to gain clearer insights here. In addition, further research is desirable, both at the theoretical-conceptual and empirical levels, to identify more precisely the specific influences of personal and social norms, and in this way contribute to theory development. This would also be particularly desirable with regard to the conceptualization of social norms (both descriptive and injunctive), where some differences are evident in the literature (cf., e.g., Thøgersen, 2006 vs. Göckeritz et al., 2010). This study has adopted Thøgersen’s taxonomy of norms (subjective social norms as a subclass of injunctive norms) and his views on important distinguishing criteria between descriptive and injunctive norms. However, especially in the case of injunctive social norms, it becomes clear that the choice of conceptualization can also result in differences in the operationalization of the constructs. In addition to the aspects discussed here in the organizational context, others such as cultural background should then be added to the conceptualization and understanding of normative influences and their modes of action. One limitation that must certainly be mentioned is the low response rate, especially among the subsample of students (i.e., 6%), which is significantly lower than expected values according to relevant studies (cf. Wu et al., 2022). Here, a self-selection effect may have played a distorting role. The voluntary nature of participation in the online survey suggests that people with a special affinity for environmental topics such as energy saving, etc., and with a higher commitment to the UdS might be overrepresented in the sample (e.g., employees showed higher commitment than students). However, the large sample size and the variance in the variables indicate a certain heterogeneity. Furthermore, the data were cross-sectional, which does not allow causal inferences. Future studies should apply a longitudinal design to further investigate this topic. Taking into account that the amount of distance learning offers increases, future studies should also take this phenomenon (students and employees which study and work remotely, i.e., at their homes) into account.

In this context, the last two of the years of the COVID-19 pandemic in particular have shown that changes in external conditions can also change usage behavior. Despite this sharp cut, the energy demand at universities declined to varying degrees, e.g., it remained the same in laboratories, while a significant drop was measured in teaching buildings (Chihib et al., 2021). The complete shift to online teaching has not only had an impact on usage behaviors in university spaces, but especially on variables such as social norm or identification with the university. Some of the students spent their study time exclusively remotely during this period, making it difficult to establish an identity-forming connection with the social university reference groups as well as the organization as a whole. With regard to commitment to organizations in times of the COVID-19 pandemic, it was found that the higher the commitment, the more likely employees are to be engaged in the organization’s interests (cf. Alshaabani et al., 2021)–such engagement could be energy-saving behavior. At the same time, exclusive use at home has already resulted in an increased awareness of energy consumption and the associated costs, which at the tax level in Germany has led to a home office flat rate to cushion these costs.

In addition, contextual conditions such as the pandemic described above, but also Russia’s war against Ukraine, have a decisive influence on the perception of the topic of energy and energy saving at the level of society as a whole. This influence can be identified at various levels. On the one hand, the topic of energy itself is becoming more salient due to daily media reporting, related fears of supply security and an increased understanding of risk in politics and society. On the other hand, this also increases the pressure on individuals and organizations to be sensitive to the massive rise in energy costs and to develop and implement short-term as well as long-term efficiency measures. German universities, for example, switched to online teaching in the winter of 2022 to save energy. On the societal level, public support for a transformation toward a sustainable energy system was found in a recent study–even across a broad political spectrum (Steffen and Patt, 2022).

For all these points, the methodological implication is the importance of longitudinal studies that can map these changes in contextual conditions. Initial studies are already providing evidence that disruptions such as the COVID-19 pandemic are affecting energy saving behavior in households (Ahmad et al., 2022). At the same time, this illustrates the relevance of studies in this area of research and teaching organizations, since educational institutions in particular have a special position due to their multiple roles as large consumers, educational space and multipliers.

## Data availability statement

The raw data supporting the conclusions of this article will be made available by the authors, without undue reservation.

## Ethics statement

Ethical review and approval was not required for the study on human participants in accordance with the local legislation and institutional requirements. The patients/participants provided their written informed consent to participate in this study. The questionnaires were submitted in advance to both the scientific and non-academic staff councils of the university, both agreed to the survey.

## Author contributions

SH and JH contributed to the conception and design of the study. SH organized the database and wrote the first draft of the manuscript. SH and TK performed the statistical analysis. SH, JH, and TK wrote sections of the manuscript. All authors contributed to manuscript revision, read, and approved the submitted version.
