# Supplementary material for: Energy saving behavior in university organizations: The value of norm constructions in a “rational choice” action model
Source: Front Psychol. 2023 Feb 21;14:1082061. doi: 10.3389/fpsyg.2023.1082061 (PMC9989172; doi:10.3389/fpsyg.2023.1082061)
Supplement: Supplementary file 1 [file Table_1.pdf]

Supplementary Material

1.1 Supplementary Table

Factor	Item		Item source and comments
	German original	English translation	
Personal Norm	PN1: Ich finde es wichtig, dass sich die Gesellschaft mit dem Thema Energie auseinandersetzt.	I think it is important for society to deal with the topic of energy.	All three items were developed specifically for the study context
	PN2: Ich finde es wichtig, dass unsere Gesellschaft etwas dafür tut, dass die Industriestaaten Energie einsparen.	I think it is important that our society does something to help the industrialized countries save energy.	
	PN3: Ich finde es wichtig, dass wir bereits unseren Kindern vermitteln, wie wichtig Energie sparen ist.	I think it's important that we communicate to our children the importance of saving energy.	
Attitude	AT1: Für mich persönlich besitzt das Thema Energiesparen eine hohe Relevanz.	For me personally, the topic of saving energy is highly relevant.	Both items were developed specifically for the study context

	AT2: Die Rolle des Energiesparens wird insgesamt stark überbewertet	The role of saving energy is greatly overrated overall. (R)	
Injunctive social norm	ISN1: Meine Kollegen/ Kommilitonen denken, dass ich Energiesparen an der UdS unterstützen soll.	My colleagues and fellow students think that I should support energy saving at the UdS.	Both items were adapted from Venkatesh et al. (2000)
	ISN2: Wichtige Personen in meinem Umfeld an der UdS denken, dass ich Energiesparen unterstützen soll.	Important people in my environment at the UdS think that I should support energy saving.	
Descriptive social Norm	DSN1: Generell wird an der UdS das Energiesparen unterstützt.	In general, energy saving is supported at the UdS.	Bothe items adapted from Venkatesh and Zhang (2010)
	DSN2: Die Universitätsleitung der UdS unterstützt das Energiesparen.	The university management of the UdS supports energy saving.	
Perceived Behavioral control	PBC1: Ob ich oder ob ich nicht Energie an der Universität spare, liegt komplett an mir.	Whether or not I save energy at the university is entirely up to me.	First four items were adapted from Armitage and Conner (1999) and three items added that were developed specifically for the study context
	PBC2: Es gibt wahrscheinlich viele Möglichkeiten für mich, Energie an der Universität zu sparen.	There are probably many ways for me to save energy at the university.	

	PBC3: Ich denke, ich habe die Fähigkeit, Energie an der UdS zu sparen.	I think I have the ability to save energy at the UdS.	
	PBC4: Wenn es komplett an mir liegen würde, bin ich davon überzeugt, dass ich zum Energiesparen an der UdS in der Lage wäre.	If it were completely up to me, I am convinced that I would be able to save energy at the UdS.	
	PBC5: Ich kann durch mein Verhalten einen wesentlichen Beitrag zum Energiesparen an der UdS leisten.	I can make a significant contribution to saving energy at the UdS through my behavior.	
	PBC6: Durch meine Entscheidungen kann ich das Sparen von Energie hier an der UdS entscheidend voranbringen.	Through my decisions, I can make a decisive contribution to saving energy here at the UdS.	
	PBC7: Durch das Ändern meines Verhaltens kann der Energieverbrauch an der UdS verringert werden.	By changing my behavior, energy consumption at the UdS can be reduced.	
Identi- fication with the organization	IO1: Wenn jemand die UdS kritisiert, fühlt sich das wie eine persönliche Beleidigung an.	When someone criticizes the UdS, it feels like a personal insult.	All six items were adapted from Mael and Ashforth (1992)
	IO2: Ich interessiere mich sehr dafür, was andere über die UdS denken.	I am very interested in what others think about the UdS.	

	IO3: Wenn ich über die UdS spreche, sage ich meistens „wir“ statt „sie“.	When I talk about the UdS, I usually say "we" instead of "they".	
	IO4: Die Erfolge der UdS sind auch meine Erfolge.	The successes of the UdS are also my successes.	
	IO5: Wenn jemand die UdS lobt, fühlt sich das wie ein persönliches Kompliment an.	When someone praises the UdS, it feels like a personal compliment.	
	IO6: Wenn die UdS in den Medien kritisiert würde, würde ich mich verlegen fühlen.	If the UdS was criticized in the media, I would feel embarrassed.	
Energy Saving Intention	ESI1: Ich beabsichtige zukünftig (weiterhin) durch mein Verhalten an der Universität dazu beizutragen, dass möglichst viel Energie eingespart wird.	In the future, I intend to (continue to) contribute to saving as much energy as possible through my behavior at the university.	Both items from Sachet (2010)
	ESI2: Ich beabsichtige zukünftig (weiterhin) meinen Energieverbrauch in Universitätsgebäuden so gering wie möglich zu halten.	In the future, I intend to (continue to) keep my energy consumption in university buildings as low as possible.	

Energy Saving Behavior	ESB1: Ich halte Türen zwischen unterschiedlich geheizten Räumen geschlossen.	I keep doors between rooms with different heating systems closed.	All nine items were developed specifically for the study context
	ESB2: Ich nutze den Energiesparmodus bei meinem PC.	I use the energy saving mode on my PC.	
	ESB3: Ich drehe die Heizung, bevor ich heim gehe, herunter, soweit dies technisch möglich ist.	I turn down the heating before I go home, as far as this is technically possible.	
	ESB4: Ich praktiziere Stoßlüften, soweit dies technisch möglich ist.	I practice shock ventilation, as far as this is technically possible.	
	ESB5: Ich mache das Licht aus, wenn ich den Raum verlasse und niemand mehr im Raum ist.	I turn off the light when I leave the room and there is nobody left in the room.	
	ESB6: Ich schalte Elektrogeräte, wenn ich sie nicht nutze, aus.	I switch off electrical appliances when I am not using them.	
	ESB7: Während des Lüftens drehe ich die Heizung herunter, soweit dies technisch möglich ist.	I turn down the heating during airing, as far as this is technically possible.	

ESB8: Ich achte darauf, dass Seminar- und Arbeitsräume nicht überhitzt werden.	I make sure that seminar and work rooms are not overheated.
ESB9: Ich achte darauf, dass keine Gegenstände vor dem Heizkörper stehen.	I make sure that no objects are placed in front of the radiator.

Supplementary Table 1. Variables, Items and Sources.

References to Supplementary Table 1

Armitage, C. J., and Conner, M. (1999). The theory of planned behaviour: assessment of predictive validity and ’perceived control’. *Br. J. Soc. Psychol. 38*, 35–54. doi: 10.1348/014466699164022

Mael, F., and Ashforth, B. E. (1992). Alumni and their alma mater: a partial test of the reformulated model of organizational identification. *J. Org. Behav. 13*, 103–123. doi: 10.1002/job.4030130202

Sachet, M. (2010). *Begriffsbestimmung und Erfassung des Konstrukts Energiebewusstsein [Definition and measurement of the construct energy awareness]*. Diploma thesis. Magdeburg: Otto-von-Guericke-Universität Magdeburg.

Venkatesh, V., Morris, M. G., and Ackerman, P. L. (2000). A longitudinal field investigation of gender differences in individual technology adoption decision-making processes. *Org. Behav. Hum. Dec. Proc. 83*, 33–60. doi: 10.1006/obhd.2000.2896

Venkatesh, V., and Zhang, X. (2010). Unified theory of acceptance and use of technology: US vs. China. *J. Glob. Inf. Tech. Manag. 13*, 5–27. doi: 10.1080/1097198X.2010.10856507
